# Effect of equilibrium pH on the structure and properties of bleach‐damaged human hair fibers

**DOI:** 10.1002/bip.23401

**Published:** 2020-09-14

**Authors:** Ernesta Malinauskyte, Paul A. Cornwell, Louise Reay, Neil Shaw, Jordan Petkov

**Affiliations:** ^1^ TRI Princeton Princeton New Jersey USA; ^2^ Lonza Global Centre of Hair Care Technology Manchester UK

**Keywords:** bleached hair, equilibrium pH, hair mechanical properties, hair protein, hair thermal properties, hair water sorption, swelling

## Abstract

Hair proteins are significantly affected by environmental pH. This impact tends to increase with prior hair damage. To understand how pH affects bleached hair properties, we utilized a number of techniques allowing for the determination of hair thermal properties, swelling and water sorption, and dry and wet tensile properties. At pH 5, hair proteins had the best structural integrity, as determined by differential scanning calorimetry and the highest tensile modulus. At pH 10, protein cross‐linking density decreased, water content and hair cross‐sectional diameter increased. Alkaline treatment, when compared with pH 5, did not reduce intermediate filament conditions (evaluated via enthalpy measurement) nor mechanical property performance in the wet state. In contrast to alkaline‐treated hair, bleached hair equilibrated at pH 3 behaved very differently: it contained two different crosslink density zones, was the least stiff in dry and stiffest in wet conditions. Additionally, it absorbed less water and had the lowest diameter because of reduced water binding by protonated carboxyl groups. The pH 3 to 10 did not affect the mechanical strength of bleached hair in dry or wet conditions.

## Graphical abstract

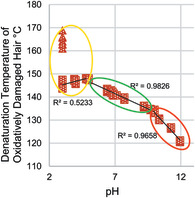

## 1 INTRODUCTION

Human hair is a complex, multi‐compartmented biological substrate. It comprises an inner cortex encased in a protective cuticle layer. The cortex makes up most of the hair mass and contains keratin and keratin‐associated proteins (KAP) that provide much of the fiber's mechanical strength. Thicker hair may also have a medulla, which consists of air‐filled voids and runs, laterally, along the center of the fiber.

The key structural proteins in the hair cortex are the keratins. Keratins are sub‐classified as acidic type I keratins and neutral type II keratins.^[^ ^1^ ^]^ Types I and II coil together in pairs, in α‐helical structures called protofilaments.^[^ ^2^ ^]^ Seven to ten pairs of protofilaments then cluster together to form intermediate filaments (IF).^[^ ^2^ ^]^ IF's are embedded in an amorphous, interfilamentous matrix comprised of KAP.^[^ ^2^ ^]^ The KAPs are cross‐linked with each other and with the keratins through disulfide bonds; these play a key role in determining the physical properties of the fiber.^[^ ^3^ ^]^ The combination of filaments embedded in a continuous matrix phase is commonly used to explain the mechanical properties of hair fibers and is termed the “two‐phase” model of hair.^[^ ^4^ ^]^ A modification introducing the third phase‐KAP crosslinks with IF affecting thermal and mechanical properties was proposed as well.^[^ ^5^, ^6^ ^]^

The proteins are amphoteric in nature and contain both acidic and basic side chains. At neutral pH's, away from proteins' isoelectric point, these groups attract each other through electrostatic forces and form cross‐links commonly referred to as salt‐links. These salt‐links contribute to the strength of the fiber. Titration experiments show that when hair is placed in an acid solution, hydrogen ions are absorbed by the hair. Between pH 7 and approximately pH 4 hydrogen ions concentration is low, thus relatively few hydrogen ions are absorbed by hair. Below pH 4 hydrogen ion absorption increases substantially.^[^ ^7^ ^]^ Studies on wool fibers show that below pH 4 there is an increase in the ease of fiber extension in a wet state.^[^ ^8^ ^]^ Similar studies on human hair fibers also show that below pH 4 (65% RH) there is a reduction in fiber elastic modulus,^[^ ^8^ ^]^ and that there is a small amount of fiber swelling.^[^ ^9^ ^]^ The protonation of carboxyl groups at low pH's replaces the salt‐linkages with hydrogen bonds thus weakening hair structures (much weaker bonds). Below pH 2 there may also be acid hydrolysis of peptide bonds that causes irreversible structural changes with concomitant fiber swelling.

In titration experiments with alkaline solutions, OH— ions are absorbed by the hair. Between pH 7 and pH 10, few hydroxide ions are absorbed.^[^ ^7^ ^]^ Above pH 10, hydroxide sorption increases markedly as the hydroxyl ion concentration is increased.^[^ ^7^ ^]^ As with low pH studies, high pH (more than pH 10) break salt‐bridges and weaken both hair^[^ ^7^ ^]^ and wool.^[^ ^8^ ^]^ In addition, hair undergoes considerable swelling at high pH, becoming more porous. Alkali hydrolysis of peptide and disulfide bonds above pH 10 weakens the hair further and causes hair damage.

Thermal analysis methods, such as differential scanning calorimetry (DSC), enable us to investigate the effects of treatments on the structural properties of hair proteins.^[^ ^10^ ^]^ Wet DSC techniques have been used to investigate the effects of various hair treatments. Data from these studies suggest that, broadly speaking, the keratin denaturation enthalpy (ΔH) is related to the structural integrity of the IFs, whereas the keratin denaturation temperature correlates with the cross‐linking density of the KAPs,^[^ ^10^ ^]^ some authors also include the impact of cross‐linking between KAP and IF in the interpretation of this parameter.^[^ ^6^ ^]^ The experiments combining short‐term hair soaking in acidic and alkaline conditions with wet DSC have shown that denaturation temperature and enthalpy vary markedly with different pH treatments.^[^ ^6^ ^]^ Data show that both measures increase as the pH is reduced from 7 to 1 and decrease as the pH is increased from 7 to 13. The same study also showed no change in virgin hair mechanical properties between pH 1 and pH 13. To some extent, this contradicts the historical evidence on wool and hair, which was discussed already above, but the differences could possibly be explained by the short duration of treatments.

The present study is part of a larger study that aims to modify environmental treatment conditions in order to improve actives penetration into hair. To achieve this, we aimed at exploring the effects of long‐term exposure to H^+^ and OH^−^ ions of bleach‐damaged hair. Wet DSC experiments were performed to investigate the effects of pH on the structural integrity of IF's and KAP's. Single fiber tensile experiments explored how pH treatments affect the mechanical properties of hair. Hair diameter and, indirectly, denaturation temperature measurements determined pH effects on hair swelling. Dynamic vapor sorption studies investigated the effect of pH treatments on water uptake into the fiber as a function of humidity.

A unique feature of all these experiments is that sufficient hydrochloric acid (HCl) and sodium hydroxide (NaOH) were added to the treatment solutions to achieve the desired equilibrium pH after 24 hours soaking with a defined level of hair damage and constant mass of hair per gram of solution. This was necessary since, as has already been explained, hair fibers absorb H^+^ and OH^−^ ions and lack of strict control over parameters may introduce inconsistency within the results.

## 2 MATERIALS AND METHODS

### 2.1 Hair samples

Medium brown, European origin, hair tresses were obtained from International Hair Importers & Products, Inc. (Glendale, New York). Hair tresses were bleached using 9% hydrogen peroxide (Sigma–Aldrich, Missouri) liquid bleach. The pH of bleaching solution was 10.2, adjusted using ammonium hydroxide (Sigma–Aldrich). Bleaching temperature and duration of one bleaching cycle was 40 °C and 20 minutes, respectively. Three bleaching cycles were performed on the hair. All of the hair was dialyzed in deionized water for 48 hours while changing water twice a day to remove residual bleach solution and bleaching by‐products from the hair. To control bleaching level reproducibility, only dialyzed hair which had denaturation temperature of 141 °C to 142 °C was selected for further experiments.

### 2.2 Other materials

HCl (2 N) and 2 N NaOH solutions were obtained from Sigma–Aldrich, and diluted to 0.5 and 0.01 N to adjust pH of solutions.

### 2.3 pH measurements

pH measurements were performed using a Sper Scientific benchtop pH‐meter. The pH meter was calibrated before measurements using commercial pH 4, pH 7 and pH 10 buffers.

### 2.4 Soaking procedure

All of the soaking solutions were prepared by titrating deionized water with HCL and NaOH. The hair to liquid ratio was 1 g:250 g. Bleached hair was soaked for 24 hours in sealed glass containers to avoid impact of CO_2_ or evaporation of HCl while constantly shaking (HY‐5 orbital shaker, Zenith Lab Inc., Brea, California) at 25 ± 2 °C. After 24 hours, all hair samples were rinsed with deionized water for 30 seconds and air‐dried at 60% relative humidity (RH).

### 2.5 Differential scanning calorimetry

Thermal properties of hair were tested using DSC2500 instrument (TA Instruments, New Castle, Delaware). Three to five replicates were prepared. Prior to the testing, hair was equilibrated at 60 ± 2% RH, 20 ± 2 °C for at least 24 hours and the instrument was calibrated using an indium standard. Approximately 10 mg of finely chopped hair was placed in a high‐pressure hermetic DSC pan together with 50 μL of DI water. The heating rate was 5 °C min^−1^.

### 2.6 Hair diameter and single fiber tensile testing studies

Prior to the testing, hair was equilibrated at 60 ± 2% RH, 20 ± 2 °C for at least 24 hours. Fibers were mounted in brass crimps and dimension measurements were performed at 60 ± 2% RH at five locations using a laser scanning micrometer (FDAS770 Fiber Dimensional Analysis System, Diastron Ltd, Andover, UK). Fifty fibers from each group were extended at 40 mm min^−1^ rate. Stress‐strain curves of fibers were recorded at 60 ± 2% RH and in a wet state using a MTT690 Miniature Tensile Tester (Diastron Ltd). Tensile parameters were calculated using the UVWin 3.6 build 3 software (Diastron Ltd, Andover, UK). Young's modulus was calculated by analyzing the linear slope.

### 2.7 Swelling experiment

This part of the study had two testing groups (n = 30). Group 1‐Hair equilibrated first at pH 5 for 24 hours, dried and equilibrated at 60 ± 2% RH, measured, then equilibrated at pH 3 for 24 hours, dried and equilibrated at 60 ± 2% RH and measured again. Group 2‐Hair equilibrated first at pH 5 for 24 hours, dried and equilibrated at 60 ± 2% RH, measured, then equilibrated at pH 10 for 24 hours, dried and equilibrated at 60 ± 2% RH and measured again. Dimension measurements were performed the same as described in hair diameter and single fiber tensile testing studies section 2.6: however, eight fiber locations were measured per hair.

### 2.8 Dynamic vapor sorption experiment

For each treated hair group an approximate 20 mg sample was introduced into dynamic vapor sorption (DVS) Intrinsic instrument (Surface measurement systems, Allentown PA) and run through the following humidity program: Initial drying at 0% RH for 24 hours; Equilibrate at 10%, 20%, 40%, 60%, 80%, 90%, 95%, 90%, 80%, 60%, 40%, 20% and 10% RH for 300 minutes. Procedure was repeated one more time to assure that data are reproducible. Average data of two such cycles were calculated and presented. The data were calculated using DVS Analysis Suite V 7.0.01.12 (Surface measurement systems, Allentown PA).

### 2.9 Statistical analysis

The tensile results were statistically analyzed using the JMP 14.0.0 analytical software. The outliers were removed using the Tukey Quartile Method (includes median). The statistically significant differences between results were evaluated using a Student *t* test at 95% confidence level. The results are presented as means ± the respective SEM.

The fiber swelling results were analyzed performing a two‐tailed paired *t* test (MS Excel, 2016). The paired sample *t* test is a statistical procedure used to determine whether the mean difference between two sets of observations is zero. The direction of the difference did not matter, thus a two‐tailed hypothesis was used for the analysis.

The Pearson correlation coefficient that measures the strength of the linear relationship between two variables was performed using MS Excel PEARSON function (2016).

## 3 RESULTS AND DISCUSSION

### 3.1 Hydrogen and hydroxide ions sorption by bleached hair

**FIGURE 1:**
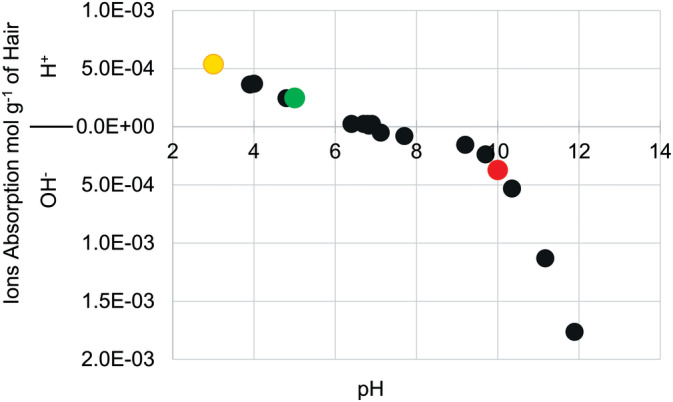
Hydrogen and hydroxide ions absorption by bleached hair treated with unbuffered HCl/NaOH solutions. HCl, hydrochloric acid; NaOH, sodium hydroxide

Figure 1 presents hydrogen and hydroxide ions absorption by bleached hair that was calculated using initial and final pH values of hair soaking solutions.

Results suggest that hair absorbs slightly more acid at pH 3 than base at pH 10 if we use the isoionic point as a reference. The isoionic point is the pH range at which the entire fiber's acid‐base side chains are at equilibrium (pH 6.2‐6.9 from our data). The starting pH of the hair after dialysis, in this study, was the isoionic point. Hair, including bleached hair, contains more carboxylic acid side chains than the basic (lysine and arginine).^[^ ^6^ ^]^ Additionally, bleached hair contains a significant amount of cysteic acid.^[^ ^6^ ^]^ The dissociation constants may help explain our findings. pK_a_ of aspartic and glutamic acids in peptides is 4.4 to 4.6.^[^ ^11^ ^]^ pK_b_ of lysine is 10 to 10.2^[^ ^11^ ^]^ and that of arginine higher than 12.^[^ ^11^ ^]^ The pK_a_ of cysteic acid is ~1.3.^[^ ^12^ ^]^ We propose that more hydrogen ions were absorbed at pH 3 than hydroxide ions at pH 10 because the protonation process of carboxylic side chains and possibly the beginning of cysteic acid protonation consumed more ions than the deprotonation process of lysine side chains.

After increasing NaOH concentration to similar levels found in relaxing treatments, the absorption increased drastically. Hair at pH 3 absorbed slightly more than twice the amount of hydrogen ions that were absorbed by hair at pH 5. Bhat *et al*., found that the rate of HCl and NaOH absorption by bleached hair is significantly higher than that of virgin hair; however, the amount of acid that bleached hair absorbs is comparable to virgin hair.^[^ ^7^ ^]^ They also found that a significant increase in the amount of absorbed hydroxide ions (NaOH) starts at a lower pH and is significantly higher than that of virgin hair. When compared to our results, the absolute values of Bhat *et al*.^[^ ^7^ ^]^ differ most likely due to different levels of bleach and the ratio between liquid volume and amount of hair, but the trends of ion absorption are comparable.

### 3.2 pH effects on thermal properties of hair

**FIGURE 2:**
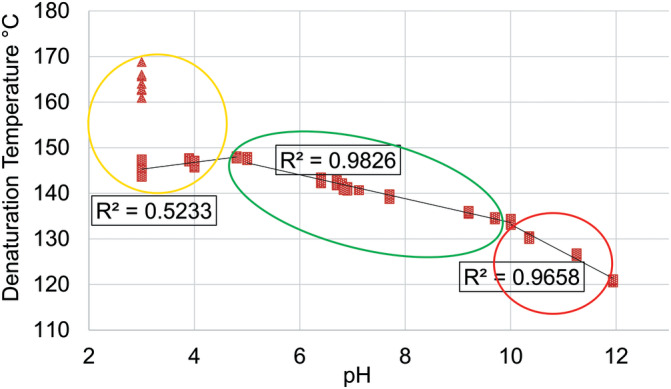
Denaturation temperature of hair treated with unbuffered HCl/NaOH solutions. HCl, hydrochloric acid; NaOH, sodium hydroxide

The denaturation temperature marks the peak of the denaturation process for the fiber's proteins. It reflects the cross‐link density of the hair matrix (amorphous protein) in which the ordered proteins are embedded.^[^ ^10^ ^]^ A summary of denaturation temperature results is presented in Figure 2.

**FIGURE 3:**
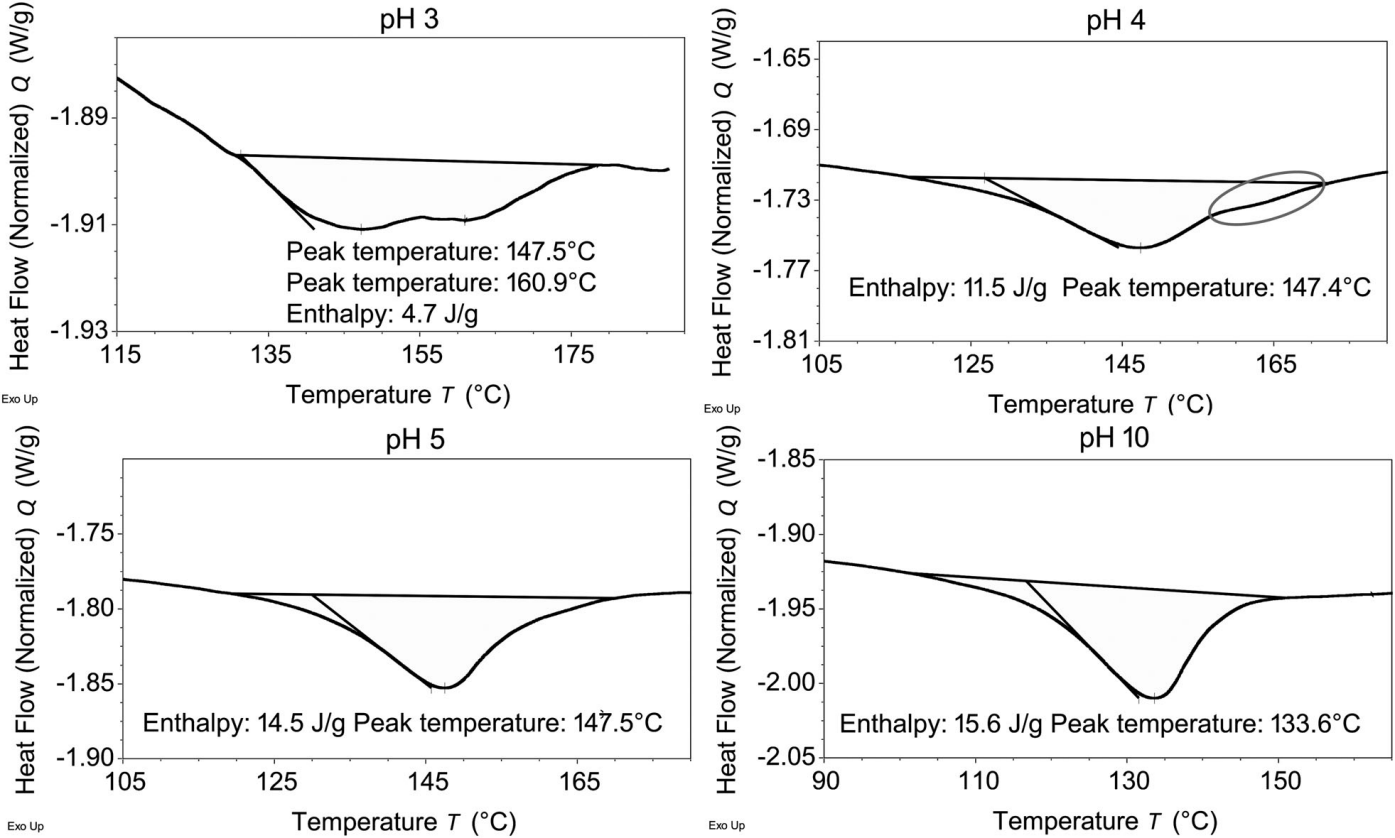
Examples of DSC traces of hair treated with unbuffered HCl/NaOH solutions. The ellipse labels the suggestion of a higher denaturation temperature peak that is already visible at pH 4. DSC, differential scanning calorimetry; HCl, hydrochloric acid; NaOH, sodium hydroxide

We observed a clear doublet of denaturation transition while testing pH 3 hair. An example of this is visualized in Figure 3 along with the single denaturation transitions of hair at pH 4, pH 5 and pH 10. Although not visible at pH 10 or pH 5, there is a clear suggestion of the second denaturation temperature peak (labeled with ellipse) at pH 4 which develops further at pH 3.

**FIGURE 4:**
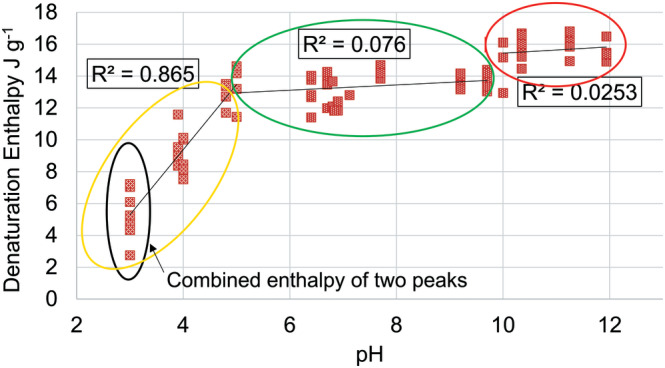
Denaturation enthalpy of hair treated with unbuffered HCl/NaOH solutions. HCl, hydrochloric acid; NaOH, sodium hydroxide

Denaturation enthalpy values reflect the relative amount and structural integrity of crystalline or ordered proteins (α helix) within a fiber.^[^ ^10^ ^]^ A summary of denaturation enthalpy results is presented in Figure 4.

In both denaturation temperature and enthalpy graphs, the trends can be represented by three straight lines between the respective pH values of lower than pH 5, between pH 5 and 10 and higher than pH 10 (Figures 2 and 4).

#### 3.2.1 pH 5 region

pH 5 region will be discussed separately as this is the pH of a typical shampoo and we chose to consider it as a typical condition of hair. At approximately pH 5, the hair proteins appear to be in the best condition: (a) denaturation temperature of 147.6 ± 0.3 °C suggests that matrix cross‐linking density is much higher than that of “neutral” hair (denaturation temperature of dialyzed hair was 142.1 ± 0.5 °C), or treated with neutral to alkaline treatments and (b) denaturation enthalpy of 13.4 ± 1.4 J g^−1^ implies that keratin crystallite condition is preserved (denaturation enthalpy of dialyzed hair was 13.6 ± 1.4 J g^−1^).

#### 3.2.2 10 > pH > 5 region (green ellipse)

In this region, a linear relationship between denaturation temperature and pH is observed. Denaturation temperature decreased with increasing pH (R^2^ = 0.98). A continuous reduction in crosslinking density can be explained via the occurrence of protonation and deprotonation processes close to dissociation constants of amino acid side chains. At pH higher than 5, systematic carboxyl group's deprotonation increased the repulsion between protein chains. Closer to pH 9, deprotonation of the lysine group possibly began. As a consequence, salt‐bridges, which had previously included lysine, were broken, which was followed by an even further increase in repulsion by available carboxyl groups.

Denaturation enthalpy results revealed that, despite a decrease in matrix stability in this region, the intermediate filament stability seemed to be preserved across the entire pH range (R^2^= 0.076 suggests denaturation enthalpy independence from pH).

#### 3.2.3 12 > pH > 10 region (red ellipse)

At pH 10, there was a shift in the steepnes os of the denaturation temperature slope. At pH 10, the basic amino acid, lysine, was neutralized to approximately 50% (according to Henderson‐Hasselbalch equation^[^ ^11^ ^]^). The results suggest this is a critical point and further deprotonation of lysine generated a “snowball” effect in repulsion between freed glutamic, aspartic and cysteic acid side chains that resulted in increasing destabilization of the matrix structure (steep slope in this region). There is a possibility that highly concentrated hydroxyl ions caused some disulfide/peptide bond cleavage as well (permanent). However, to confirm this, further research is required.

In the range of pH 10 to 12, a sudden, yet slight increase in denaturation enthalpy is observed between approximately pH 9.8 and 10. Then, above pH 10, the denaturation enthalpies plateaued at their increased position. It is quite difficult to explain the slight increase in denaturation enthalpy with the matrix being so destabilized. Similar denaturation enthalpy behavior was noticed during unrelated studies performed at TRI when bleached hair was significantly affected by UV: denaturation temperature decreased to approximately 120 °C; however, denaturation enthalpy was as high as that of UV untreated bleached hair. This may be influenced by other thermal process(es) that happen from 95 °C to 120 °C which contribute to the energy associated with protein denaturation.

#### 3.2.4 5 > pH ≥ 3 region (yellow ellipse)

In the range of pH 3 to 5, with increasing solution acidity, the single denaturation peak gradually changed into two partially separated peaks with joint denaturation enthalpy decreasing (flattening and widening of denaturation peaks was observable as well). This would suggest that part of the matrix in higher acidity samples appeared to be less dense, while the other part was significantly denser than that of hair at pH 5. We postulate that more intense matrix changes should happen closer to the surface where the hair became more compact. This possibly slowed down further penetration of HCl, which would leave the middle of the cortex less affected. In trying to explain the increasing crosslinking density of the matrix, again we hypothesize that at pH lower than 5 protonation of glutamic and aspartic acids occurred resulting in a reduction of the repulsion between negatively charged groups as well as salt‐bridges being replaced by hydrogen bonds. These processes enabled more compact packing within areas of hair represented by the higher denaturation temperature peak. Our data show that the appearance of a second denaturation temperature peak was already visible at ~ pH 4. At pH 3, the matrix still consisted of two peaks, implying that the transition was not completed.

The combined denaturation enthalpies of both peaks are plotted in Figure 3. Deconvolution of these peaks could help to clarify the ratio of peak one and peak two energetics, although, because the enthalpies are so low it may not change the result of parameters correlation. Therefore, we will leave this task for future investigation. Although the matrix cross‐linking density increased, it seems that the lower energy of hydrogen bonding made it easier to dismember the matrix, thus, the total energy of denaturation diminished. Additionally, because bleached hair before pH treatments are weakened further by the bleach, acidic peptide hydrolysis is also possible. We expect signs of hydrolysis to be expressed in the wet state of tensile performance where water could help to reveal such effects.

The literature analysis showed very limited research being done on pH effect on hair thermal properties. Istrate *et al*. concentrated researching on virgin hair property changes and only few acidic pH points were dedicated to the bleached hair. The authors found an increase in denaturation temperature and enthalpy^[^ ^6^ ^]^ which partially contradicts our findings. The differences in the acidic region could be explained by the following differences in the treatment protocol (Istrate *et al*.^[^ ^6^ ^]^ vs Malinauskyte *et al*.): (a) different bleaching level; (b) bleached hair was rinsed with water versus dialysis which allowed the complete removal of prior alkaline treatment from the cortex; (c) treatment of 30 minutes vs 24 hours treatment; (d) acetic acid was used to lower the acidity of the solution versus HCl; (e) post treatment included few minutes rinse and overnight dialysis as opposed to 30 seconds rinse; (f) it is unclear if pH varied during 30 minutes soak versus equilibrium pH was used. As there were at least six different variables, it is difficult to answer which of these could have a major impact on the differences between our results.

### 3.3 pH effects on hair water sorption properties

**FIGURE 5:**
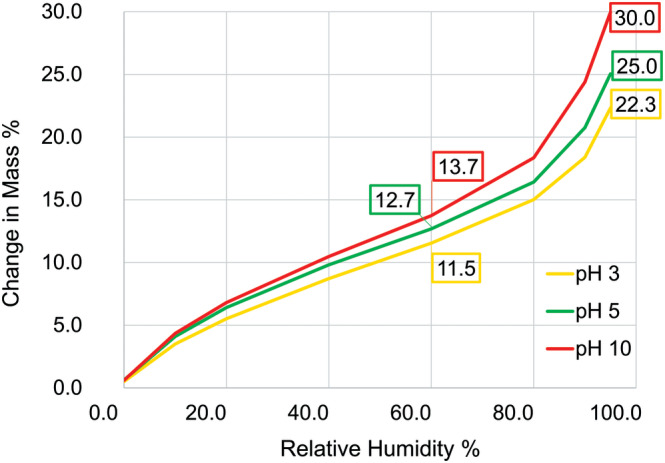
Water desoption properties of hair treated with unbuffered HCl/NaOH solutions. HCl, hydrochloric acid; NaOH, sodium hydroxide

All mechanical and dimensional measurements were performed after drying fibers naturally from the wet state, thus, for this analysis we selected the water desorption isotherm. Water desorption results are presented in Figure 5.

From the 60% RH data, it is clear that the differences between pH 3 and pH 5; and pH 5 and pH 10 are rather small. At 95% RH, these differences increased significantly. Interestingly, pH 3 hair absorbed approximately 26% less water than pH 10 hair. The literature is quite limited regarding pH effect on water sorption of hair. Breauer and Prichard found that at pH lower than 2, the absorption of reducing agents by hair decreased,^[^ ^13^ ^]^ which agrees with our observations using water as a penetrant. The authors investigated whether abnormalities in the sorption of reducing agents could be caused by peptide or disulfide bond hydrolysis. Amino acid end groups and amino acid analysis did not show evidence that such structural changes would have occurred while virgin hair was soaked at pH lower than 2 solution.^[^ ^13^ ^]^

However, observed differences in water sorption at different pH can be explained via differences in side chain solvation by water or hydration. The hydration number is defined as the average number of water molecules that are bound stronger to the compound of interest than to other water molecules. The papers that studied hydration of amino acids at different pH suggest the following: (a) hydration number at pH 4 (below pK_a_) for aspartic acid and glutamic acid is approximately two^[^ ^14^ ^]^ to three^[^ ^15^ ^]^ times lower than at pH 6 to 8 (above pK_a_)^[^ ^16^ ^]^; (b) hydration numbers of protonated glutamic and aspartic acids are lower than that of lysine and arginine;^[^ ^14^, ^15^ ^]^ (c) hydration numbers of charged carboxylic side chains of glutamic and aspartic amino acids are higher than those of lysine and arginine.^[^ ^15^, ^16^ ^]^ These findings fit our results and help to explain the trends we observed. Additionally, these also can be used to explain the effect of other acids and bases on hair.

### 3.4 pH effects on hair swelling properties

Cross‐sectional area with the measured minimum and maximum diameter of hair soaked in different pH solutions are presented in Tables 1 and 2, respectively. All measurements were performed at 60% RH.

**TABLE 1:** Dimensional parameters' summary of fibers that were soaked in pH 5 and pH 3 solutions

Group	pH 5		pH 3		Difference (%) in comparison to pH 5	
	Cross‐sectional area (μm^2^)	Mean diameter (μm)	Cross‐sectional area (μm^2^)	Mean diameter (μm)	Cross‐sectional area (μm^2^)	Mean diameter (μm)
1	2561.5	59.3	2516.9	58.7	−1.7	−1.0
2	3617.2	71.4	3492.8	70.6	−3.4	−1.2
3	5320.6	86.8	5155.2	84.8	−3.1	−2.3
Total	3833.1	72.5	3721.6	71.4	−2.9	−1.6

**TABLE 2:** Dimensional parameters' summary of fibers that were soaked in pH 5 and pH 10 solutions

Group	pH 5		pH 10		Difference (%) in comparison to pH 5	
	Cross‐sectional area (μm^2^)	Mean diameter (μm)	Cross‐sectional area (μm^2^)	Mean diameter (μm)	Cross‐sectional area (μm^2^)	Mean diameter (μm)
1	2524.7	58.0	2580.6	58.8	2.2	1.3
2	3500.6	67.5	3533.5	68.2	0.9	1.0
3	5068.4	83.3	5119.6	84.7	1.0	1.6
Total	3697.9	69.6	3744.5	70.6	1.3	1.4

The average change in fiber dimensions after soaking pH 5 fibers in pH 3 solution is presented in the “Total” row. The difference in cross‐sectional area and mean diameter is rather small (−2.9% and−1.6%, respectively). Two‐tailed paired *t* test was performed to evaluate whether there is a difference, on average, in the fiber dimensions between “before” pH 5 and “after” pH 3. The null hypothesis (H_0_) assumed that the true mean difference is equal to zero. The two‐tailed alternative hypothesis (H_1_) assumed that true mean difference is not equal to zero. The calculation revealed that *P* = .002. Consequently, the decrease in fiber dimensions is significant at the α = 0.01 level.

To observe if the initial fiber thickness was important to the swelling of the hair, we sorted the fibers according to their cross‐sectional area in three groups. Grouped by size of cross‐sectional area, results (Table 1) showed trends of reduction in fibers dimensions after soaking hair in pH 3 solution: (a) Approximately 1% decrease in minimum and maximum diameters and 1.7% decrease in cross‐sectional area was observed in the finest fibers (average of 2562 μm^2^ cross‐sectional area); (b) slightly over 3% decrease in cross‐sectional area, ~ 2% decrease in maximum diameter, and 1% to 1.8% decrease in minimum diameter was observed when testing thicker fibers (groups of 3616 and 5321 μm^2^ cross‐sectional area). Decreased diameter could be explained by a reduced number of negatively charged amino acids that cannot repel each other and are less hydrated.

Different trends were found in how different thickness of hair is affected by alkaline pH (Table 2).

The average change in fiber dimensions after soaking pH 5 fibers in pH 10 solution is presented in the “Total” row. The difference in cross‐sectional area and mean diameter is rather small (1.3% and 1.4% respectively). Two‐tailed paired *t* test was performed to evaluate whether there is a difference, on average, in the fiber dimensions between “before” pH 5 and “after” pH 10. The calculation revealed that *P* = .00003. The increase in fiber dimensions is significant at the α = 0.01.

Thinner hair swelled more than thicker hair. The cross‐sectional area of the finest hair (2525 μm^2^) increased by little over 2%, while the cross‐sectional area of both thicker hair groups increased by ~ 1%.

Besides general knowledge that hair swells above the isoelectric point and allows reducing agents/dye/bleach penetration into hair,^[^ ^17^ ^]^ we could not find published data regarding bleached hair swelling due to exposure to different pH solutions. The reduction in fiber diameter at pH 3 and its increase at pH 10, in comparison to pH 5 hair, was similar to that of virgin hair reported previously.^[^ ^18^ ^]^

### 3.5 pH effects on mechanical properties of hair

The literature suggests that DSC and tensile parameters correlated well while studying bleached and thermal damage levels.^[^ ^19^ ^]^ These treatments permanently changed the hair structure. pH treatment, excluding extreme values, cosmetically modified hair proteins (recoverable changes), thus, it is useful to understand if (a) large observed changes in hair matrix crosslinking and (b) integrity of IF across the pH range, can translate into significant changes in the mechanical properties of hair. Due to tensile experiments being labor intensive and costly, we decided not to continue with the property investigation across the entire pH range, but to select consumer relevant pH representatives from green, yellow and red ellipse zones. pH 3 corresponds to acidic treatments such as lemon juice, vinegars or certain patented hair care formulations.^[^ ^20^ ^]^ pH 5 is a conventional pH for most hair care products (for this reason, pH 5 hair will be treated as the baseline). pH 10 is a common pH in hair coloring systems.

#### 3.5.1 Dry tensile test results

**FIGURE 6:**
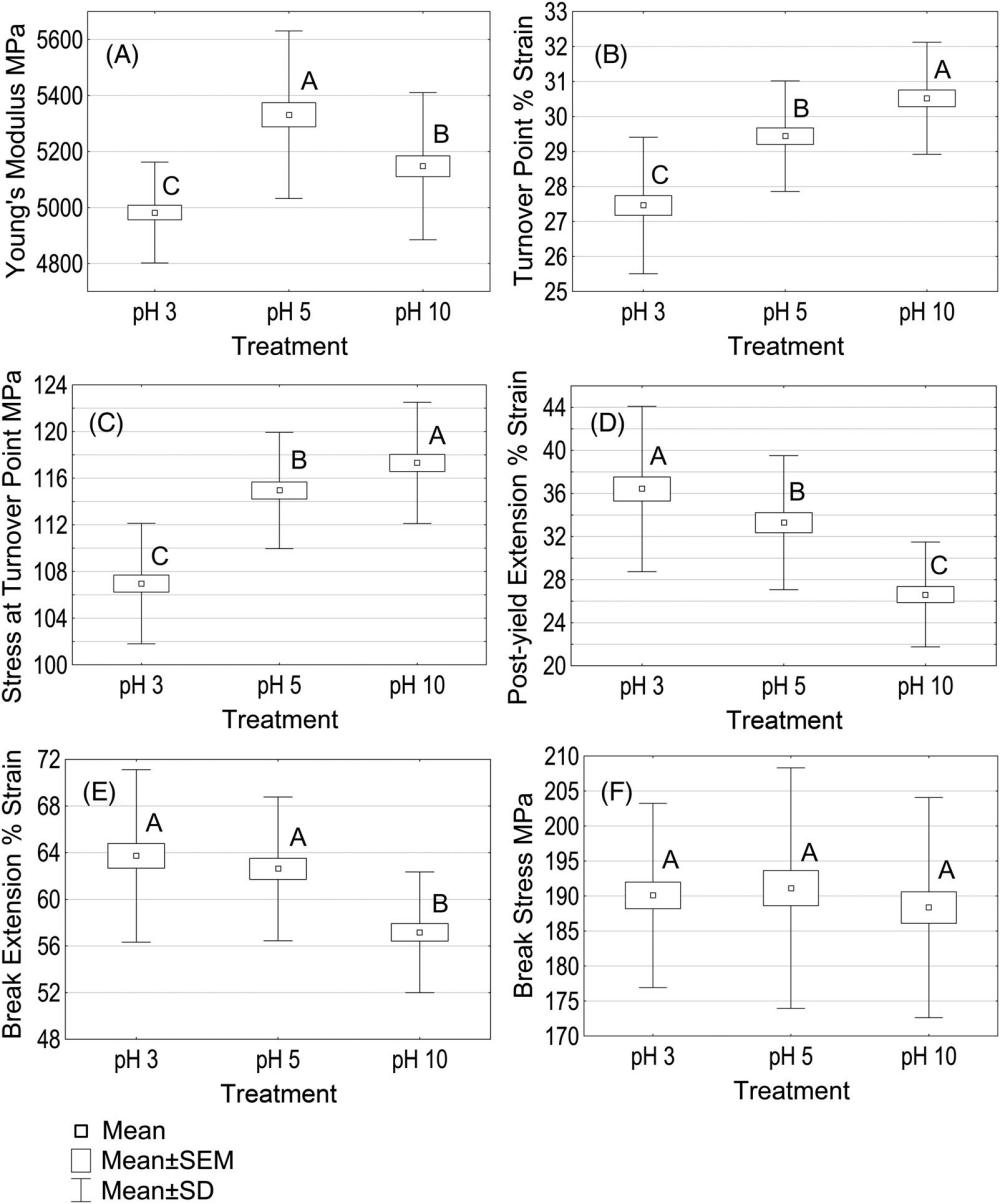
Dry tensile properties of hair treated with unbuffered HCl/NaOH solutions. Data not connected by the same capital letter are statistically different (95% confidence level): A, Young's modulus; B, Turnover point extension; C, Stress at turnover point; D, Post‐yield extension; E, Break extension; F, Break stress. HCl, hydrochloric acid; NaOH, sodium hydroxide

Dry Young's modulus, turnover point and its stress, post‐yield extension, break stress and break extension data are presented in Figure 6.

The majority of models explaining hair stress/strain behavior agree that, in the linear region, the stretching of chemical bonds takes place.^[^ ^4^, ^21^, ^22^ ^]^ This means that, depending on the dominance of particular bonds, we may observe different behavior. By controlling the denaturation temperature of the samples prior to pH treatment, we are safe to assume that the level of disulfide bonds is similar among different treatment groups. Therefore, by varying environmental pH, we aim to evaluate the importance of the ratio between salt‐bridges and hydrogen bonds, and repulsion between free negatively charged groups on the mechanical properties of bleached hair. The highest Young's modulus was obtained when measuring pH 5 hair, followed by pH 10, then pH 3. These differences, at the 60% RH, were found to be statistically significant. We assume that at pH 5 there is a favorable ratio between salt‐bridges and hydrogen bonds, and at the same time, lower repulsion (than that of pH 10 hair) between side chains was achieved which contributed to pH 5 hair resistance to recoverable deformations. Meanwhile, the lower Young's Modulus of pH 3 hair could be explained by the highest number of hydrogen bonds and the lowest number of salt‐linkages: the hydrogen bond is significantly weaker than salt‐linkage, thus the stretching of the hair required less load per length unit. We were unable to find dry tensile data for bleached hair soaked at different pH. To the best of our knowledge, only Bhat *et al*. studied the viscoelastic properties of dry (65% RH) virgin hair.^[^ ^7^ ^]^ Similar to our results, they found that the dry state elastic modulus decreased at both pH extremes. However, a larger decrease in modulus was found in alkaline treated hair.^[^ ^7^ ^]^ The differences could possibly be attributed to differences in initial hair condition and their pH range was one pH unit more extreme.

Results suggest that pH affected turnover point stress and strain of bleached hair fibers as well. Both parameters of pH 3 soaked hair were the lowest, and those of pH 10 treated hair were the highest. pK_a_ of the thiol group is 8.5 to 8.8.^[^ ^11^ ^]^ We anticipate that further research of thiol/disulfide interchange (happens above pK_a_) under these pH could help explain the observed differences in these results.

When analyzing hair resistance to tensile stress, it is clear that pH 10 hair had the shortest extension in the post‐yield region (20% and 27% in comparison to pH 5 and pH 3 hair) which significantly contributed to the total shortness of break extension. Explaining the significance of pH 10 hair having higher resistance to deformations in the post‐yield region is difficult. Publications are contradictory as to what is happening in this region while testing virgin hair. The situation with chemically treated hair is worse: currently there is no model, which would properly interpret chemically or UV damaged hair responses to deformations in the post‐yield region.^[^ ^23^ ^]^ Although the correlation of dry break extension with denaturation enthalpy might provide some clues, our interpretation as to why pH 10 hair was more resistant to deformations in the post‐yield region may be illuminated by current ongoing research focused on developing such a model.

The analysis revealed no pH impact on dry hair break stress.

#### 3.5.2 Wet tensile test results

**FIGURE 7:**
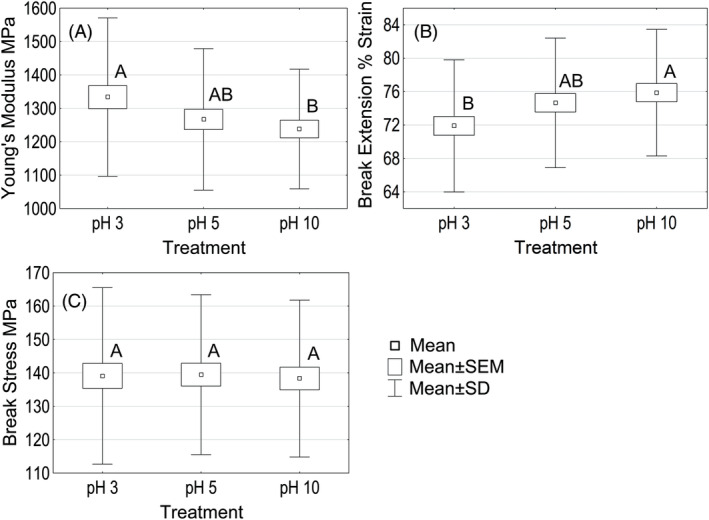
Dry tensile properties of hair treated with unbuffered HCl/NaOH solutions. Data not connected by the same capital letter are statistically different (95% confidence level): A, Young's modulus; B, Break extension; C, Break stress. HCl, hydrochloric acid; NaOH, sodium hydroxide

Chemically damaged hair was especially weakened by water. Plasticization by water helps to amplify the level of damage, which would be hardly measurable in dry conditions. By completing this experiment, we hoped to not only assess the IF condition but also to investigate if potential peptide bond hydrolysis is measurable. While comparing wet and dry tensile profiles, we found a 4‐fold reduction in stresses that was required to reach the turnover point in a wet state, and the break‐stress in the wet state was approximately 35% lower than that in a dry state. A truly linear region was found at less than 1% strain extension. This suggests that the matrix contribution to the hair's mechanical properties diminished. Wet Young's modulus, break stress and break extension data are presented in Figure 7.

Clearly, hair equilibrated at pH 5 shared similarities with hair that was equilibrated at pH 3 or pH 10 due to water breaking the majority of salt‐bridges and hydrogen bonds that were responsible for differences observed in a dry state. Istrate *et al*. tested only acidic pH treated bleached hair and reported no changes in wet tensile properties between pH 3 and pH 5 as well.^[^ ^6^ ^]^ Hair at pH 3 had a significantly higher Young's modulus and resistance to length deformations than those of pH 10 treated hair. Our water sorption study showed a large difference in pH 3 and pH 10 water sorption at 95% RH. Matrix cross‐link density and protein solvability changes may have impacted the water sorption by fibers and the difference in hydration/plasticization was possibly significant enough to cause a measurable difference in pH 3 and pH 10 hair properties.

Similarly, to the dry tensile break stress results, no statistically significant difference was found when comparing hair at pH 3 or pH 5 with pH 10 hair. If peptide bonds hydrolysis occurred during hair soaking at low pH, it was possibly masked by reduced water sorption in pH 3 hair, thus, not measurable even at the weakest (wet) state of hair.

### 3.6 Correlations between parameters

Comparing the extent of changes in measured properties, large changes due to pH effect in DSC results have not translated into large changes in mechanical properties. The analyses showed that the stresses that are required to generate breakage were very similar amongst differently treated hair. This means that pH induced changes could only be observed while analyzing the behavior differences in elastic and post‐yield regions, and the break extension. The Pearson correlation coefficients between the tensile, DSC and water gain parameters at pH 3, pH 5 and pH 10 are presented in Table 3.

**TABLE 3:** Dimensional parameters' summary of fibers that were soaked in pH 5 and pH 10 solutions

	Lower denaturation temperature peak at pH 3	Higher denaturation temperature peak at pH 3	Denaturation enthalpy	Water gain at 60% RH	Water gain at 95% RH
Dry Young's modulus	0.21	−0.53	0.81	0.49	—
Dry break extension	0.94	0.91	−0.68	−0.93	—
Wet Young's modulus	0.60	0.99	−0.97	—	1.00
Wet break extension	−0.57	−0.98	0.98	—	−1.00
Lower denaturation temperature peak at pH 3	—	—	—	—	−0.86
Higher denaturation temperature peak at pH 3	—	—	—	—	−0.97
Denaturation enthalpy	—	—	—	—	0.81

Abbreviation: RH, relative humidity.

Decent positive correlations were found between dry Young's modulus and denaturation enthalpy; denaturation enthalpy and 95% water gain (0.81). Strong positive correlation (higher than 0.90) was determined when analyzing: (a) dry break extension versus denaturation temperature (denaturation temperature both peaks); (b) wet Young's modulus versus denaturation temperature (with higher pH 3 peak) and versus water gain at 95% RH; (c) wet break extension versus denaturation enthalpy; (d) denaturation enthalpy versuss water gain at 95% RH.

Strong negative correlation (lower than−0.90) was found when comparing: (a) wet Young's modulus versus denaturation enthalpy; (b) wet break extension versus denaturation temperature (with higher pH 3 peak) and versus water gain at 95% RH; (c) dry break extension versus 60% RH water gain; (d) water gain versus both denaturation temperature peaks.

Despite parameters correlating well among each other, it is well known that correlation does not mean proof. To investigate which correlations can be used for the prediction of fiber property response to different compounds, further research is required.

## 4 CONCLUSIONS

Bleached hair thermal, tensile, water sorption and swelling properties are affected by the pH. The effect levels for each property differs. Varying the pH changes the protonation and deprotonation ratio in amino acid side chains, which in turn affects the ratio of salt bridges and hydrogen bonds, and protein solvation. These affect thermal properties, water sorption and dry tensile properties the most. Water interferes with hydrogen and electrostatic bonds, therefore, wet tensile properties are affected by pH less. The break stress at wet or 60% RH is not affected by pH 3 to 10, which suggests that even if peptide hydrolysis happens at some extent in hair, the relatively small damage level is not measurable by tensile testing. The analysis of parameters correlation suggests that bleached hair properties changes systematically together with protonation or deprotonation of amino acid side chains. Our findings not only fill current gap in scientific knowledge regarding the relationship between thermal, mechanical and water sorption properties of bleached hair, but could also open new opportunities applying this knowledge into manipulation of hair properties using other compounds as well. For example, the increase in water sorption and diameter and the decrease in the cross‐linking density at pH 10 suggest that pH higher than 10 is the best range to facilitate the penetration of actives. Future studies will consider this postulation by examining the penetration of small‐labeled molecules into the fibers.

## CONFLICT OF INTEREST

The authors declare no competing interests. Lonza and TRI jointly agreed the design of the study; in the collection, analyses, and interpretation of the data, in writing and reviewing of the manuscript, and in the decision to publish the results.

## Data Availability

The data that support the findings of this study are openly available in TRI Library at https://library.triprinceton.org/1erqg3t/ (bottom of the page), reference ID 1erqg3t.
